# Immunological routine laboratory parameters at admission influence the improvement of positive symptoms in schizophrenia patients after pharmacological treatment

**DOI:** 10.3389/fpsyt.2023.1082135

**Published:** 2023-03-22

**Authors:** Anna Skalniak, Wirginia Krzyściak, Natalia Śmierciak, Marta Szwajca, Paulina Donicz, Tamas Kozicz, Maciej Pilecki

**Affiliations:** ^1^Department of Endocrinology, Faculty of Medicine, Jagiellonian University Medical College, Kraków, Poland; ^2^Department of Medical Diagnostics, Faculty of Pharmacy, Jagiellonian University Medical College, Kraków, Poland; ^3^Department of Child and Adolescent Psychiatry, Faculty of Medicine, Jagiellonian University Medical College, Kraków, Poland; ^4^Department of Clinical Genomics, Mayo Clinic, Rochester, MN, United States

**Keywords:** schizophrenia, inflammation, laboratory diagnostic tests, outcome of a patient’s treatment, cost optimization in psychiatry

## Abstract

**Introduction:**

The standard care of schizophrenia patients is based on the assessment of their psychotic behavior, using interview-based, subjective scales that measure symptoms severity. We aimed at defining easily accessible and inexpensive blood-derived clinical diagnostic parameters that might serve as objective markers in the prediction of the effects of pharmacological treatment of schizophrenia patients.

**Methods:**

A total of 40 patients with schizophrenia diagnosis according to ICD 10 during psychotic decompensation were included in the study. Blood-based biochemical parameters, BMI and interview-based medical scales of symptom severity were determined – all at admission and after 12 weeks of standard pharmacological treatment.

**Results:**

The drops in scale values were correlated with clinical parameters. All scale changes after treatment were dependent on the value of the given scale at admission, with higher initial values leading to larger drops of the values after treatment. Models based on those correlations were significantly improved when immune and metabolism parameters were included. C4 complement and C-reactive protein (CRP) level at admission were predictive of changes in Positive and Negative Syndrome Scale (PANSS) subscales related to significant disruption of thought processes, reality testing and disorganization. The pharmacological treatment-driven changes in scales representing negative symptoms were correlated with markers of the patients’ thyroid status and metabolism.

**Discussion:**

We show that objective markers can be obtained by testing immune and metabolic parameters from the patients’ blood and may be added at a low cost to the standard care of schizophrenia patients in order to predict the outcome of pharmacological treatment.

## 1. Introduction

The immune system has been known to be involved in the pathophysiology of schizophrenia for many years, and it is also known that the immune answer is being modified by antipsychotic therapy (1, 2). Lately, the interest in the involvement of infections and the immune system in schizophrenia has received growing interest. Research has proven that prenatal exposure to microbial infections may be an environmental factor in schizophrenia development (3, 4), and viral infections during childhood might trigger psychoses and relapses of schizophrenia (5). It has been shown that the acute psychotic state is characterized by increases in immune markers and normalizes after treatment (6). Also, the fact that treatment with anti-inflammatory drugs shows beneficial effects on the symptomatology of schizophrenia indicates that immune dysfunction in schizophrenia is related to the pathomechanism of the disorder (7, 8).

In a study which evaluated the proinflammatory state in the first episode of schizophrenia, IL-6 was recognized as a state marker for acute exacerbations and tumor necrosis factor alpha (TNFα) as a trait marker of schizophrenia (9). However, in yet another study, there was no difference in levels of IL-6 and TNFα in exacerbation compared to remission in schizophrenic patients. IL-6 was, however, higher and TNFα was lower in schizophrenic patients in both exacerbation and remission in comparison with healthy controls (10). A recent meta-analysis revealed an association of schizophrenia risk with genetic variants in the promoter region of TNFα (11). At the same time, it was noted that there was an association between symptoms of disease severity with a number of peripheral pro-inflammatory cytokines and salivary microflora in patients with a first psychotic episode. Bacteroidetes, Thermomicrobia, and Haemophilus showed negative associations with C-reactive protein (CRP) levels. In contrast, pathogenic Aggregatibacter, Campylobacter, Fusobacterium, Haemophilus, and Veillonella showed negative correlations with interferon gamma (IFNγ), interleukin-8 (IL-8), and/or TNFα levels (12). The intestinal microflora, by regulating the activity of pro-inflammatory cytokines or bacterial antigens of different microbial species, can influence the function of the hypothalamic-pituitary-adrenal axis mainly related to cortisol release and modulation of cognitive function (13, 14).

Pro-inflammatory serum cytokines including IL-6 and IL-17 are elevated in patients after with schizophrenia pharmacological treatment (15), and have been shown to be deregulated in treatment-resistant schizophrenia (16, 17). Research on cytokines has also revealed that the Positive and Negative Syndrome Scale (PANSS) cognitive subscore is correlated with the duration of illness and cortisol together with TNFα and IL-8 serum values (18). Upon treatment with the anti-inflammatory drug ibuprofen, however, it was seen that TNFα levels secreted by peripheral blood mononuclear cells from schizophrenic patients were significantly lower than from controls. IL-10 production correlated positively with the PANSS positive subscale score (19).

However, while a link between psychiatric disfunctions and the immune system has been indicated in a growing number of investigations, this does not reflect the existence of a specific direct causal link. Other variables, like neurobiological dysfunctions, may also be involved in the pathophysiology of those diseases (20). Due to the increased risk of suicidal behavior related to depressive symptoms and unsuccessful therapeutic responses, there is a need for novel strategies to identify non-respondents to pharmacotherapy, who would benefit from additional therapies. While novel strategies for the treatment of psychiatric states are emerging (21), there is a growing need to develop prediction strategies in order to minimize ineffective treatment in patients who do not respond, or do only partially respond to standard pharmacotherapy.

The role of the immune system in the pathomechanism of schizophrenia is therefore well-supported in literature data. There is, however, still a lack of clear indications about how to use this knowledge in the everyday routine.

We aimed at assessing the correlations of routinely measured laboratory blood tests at admission with changes in psychological symptoms after unified pharmacological treatment of schizophrenia patients. Those results could enable us to make assumptions about the expected outcome of a patient’s treatment, based on easy-to-obtain and relatively inexpensive diagnostic tests performed at admission.

## 2. Materials and methods

### 2.1. Participants of the study

Forty patients were included in the study, who have been diagnosed with paranoid schizophrenia during psychotic decompensation according to the ICD-10 classification. One patient was excluded due to extremely high white blood cell values at admission, which were identified as outliers in all analyses. Among the remaining patients, 18 (46.15%) were female and 21 (53.85%) were male. The median age was 18 years, with the youngest patient aged 15 and the oldest 38 (IQR 17–29 years).

Inclusion criteria: Age between 15 and 40 years, psychical state requiring psychiatric hospitalization, medical diagnosis of paranoid schizophrenia, F20, informed consent to participate in the study.

Exclusion criteria: Inability to express informed consent, intellectual disability, hospitalization without the patient’s consent, drug or psychoactive substances abuse within 3 months before admission, overuse of alcohol or other substances (other than tobacco), previous head injuries with loss of consciousness, alcohol addiction, mental disability of the parent in the case of minor patients, psychiatric diagnosis of affective disorders (F30–F39), use of drugs (antibiotics, non-steroidal anti-inflammatory drugs, corticosteroids, vitamin preparations, and antioxidants) in the last 2 weeks before admission, presence of acute or chronic diseases: autoimmune diseases, acute inflammatory diseases, active or previous oncological diseases, chronic terminal disease, cardiovascular disorders, history of thyroid dysfunction, diabetes, and history of CNS disorders. In addition, the study excluded patients taking drugs that may affect the concentration of thyroid hormones and their precursors: oral contraceptives, estrogens, phenytoin, anti-thyroid drugs (e.g., propylthiouracil), lithium, propranolol, glucocorticoids, mineralocorticoids, anti-epileptic drugs, non-steroidal anti-inflammatory drugs, and other immunomodulation therapies.

Demographic and clinical data were collected from each patient; they were also asked to complete questionnaire surveys. Blood samples were collected during routine collection of material in the first week after the admission of the participants, as well as after 12 weeks of pharmacological treatment, each time after overnight fasting of the patients. Routine blood tests included blood count, lipid profile [triglycerides (TG), total cholesterol (TC), high-density lipoprotein (HDL), and low-density lipoprotein (LDL)], CRP, complement C3 and C4, ionogram (K+, Na+, and Mg+), glucose, creatinine, cortisol, and thyroid panel (fT3, fT4, and TSH). Routine laboratory tests were carried out on the day of blood collection at the clinical hospital laboratory in Krakow using the automatic Sysmex XN-2000 analyzer (Cobe, Japan) for blood count and the Cobas 6000 and Cobas 8000 biochemical analyzers (Roche Diagnostics, Mannheim, Germany) to assess biochemical and hormonal parameters. The BMI was calculated based on the height and weight parameters from 3 sets of values.

Pharmacotherapy was prescribed and continued in all patients. Both classical neuroleptics and second-generation neuroleptics were used in accordance with the guidelines of the American Psychiatric Association (22). Drug doses were converted to chlorpromazine equivalents, which are defined to be a drug dose that would correspond to 100 mg of orally administered chlorpromazine (23). The equivalent of 200–300 mg of chlorpromazine is considered a minimally effective dose, while more than 1,000 mg of chlorpromazine is considered high (24). None of the patients experienced side effects.

The study protocol has been approved by the Bioethics Committee of the Jagiellonian University, consent number: 122.6120.23.2016 from the 23rd June 2016 and 1072.6120.152.2019 from the 27th June 2019. Each study participant and their legal caretaker in the case juvenile patients gave their informed consent to become included in the study. Patients were recruited from January 2019 to January 2020.

The Positive and Negative Syndrome Scale for Schizophrenia (PANSS) (25) measures the severity of the core symptoms of schizophrenia and is considered the gold standard for psychometric assessment of schizophrenia (26). The PANSS is a semi-structured interview based on information from the past week, with 30 items on a continuum from 1 to 7 points. In a non-discrete study, we used a 5-factor PANSS model (27) consisting of the following scales: Negative symptoms, Positive symptoms, Disorganized thoughts, Uncontrolled hostility/excitement, and Anxiety/depression.

The following subscales of the PANSS scale, according to Marder et al. (27), were analyzed: PANSS_pos [“positive symptoms”: delusions (P1), hallucinatory behavior (P3), grandiosity (P5), suspiciousness/persecution (P6), stereotyped thinking (N7), somatic concern (G1), unusual thought content (G9), and lack of judgment and insight (G12)], PANSS_neg [“negative symptoms”: blunted affect (N1), emotional withdrawal (N2), poor rapport (N3), passive/apathetic social withdrawal (N4), lack of spontaneity (N6), motor retardation (G7), and active social avoidance (G16)], PANSS_dis [“disorganized thoughts”: difficulty in abstract thinking (N5), mannerisms/posturing (G5), disorientation (G10), poor attention (G11), disturbance of volition (G13), preoccupation (G15), and conceptual disorganization (P2)], PANSS_exc [“uncontrolled hostility/excitement”: excitement (P4), hostility (P7), and uncooperativeness (G8)], PANSS_emo [“anxiety/depression”: anxiety (G2), guilt feelings (G3), tension (G4), and depression (G6)]. Each of the subscales was measured at admission and after 12 weeks of unified pharmacological treatment.

The commonly used self-report State-Trait Anxiety Inventory (STAI) (28) in Polish adaptation (29) was used to assess anxiety levels. In the present study, we used the STAI-state subscale consisting of 20 items assessing the severity of anxiety at the time of the survey.

The Beck Depression Inventory – Second Edition (BDI-II) (30) in Polish adaptation (31) is a widely used self-report inventory measuring depression severity. The BDI-II contains 21 items; each item examines the presence and severity of one specific symptom of depression in the past 2 weeks on a four-point scale from 0 to 3. A higher score is indicative of higher depressiveness.

The Calgary Depression Scale for Schizophrenia (CDSS) (32), a Polish adaptation (33), is a recommended scale for measuring the severity of depressive symptoms in people with a diagnosis of schizophrenia. It distinguishes depression from the positive and negative symptoms present in schizophrenia (34, 35). The CDSS is a questionnaire consisting of 9 items with a global score range of 0–27 points. The PANSS and CDSS were completed by experienced clinicians in working with people diagnosed with schizophrenia.

The standardized versions of the STAI measure of state anxiety (STAI_state), Beck Depression Inventory (BDI) and the CDSS (Calgary) were measured for changes after 12 weeks of unified pharmacological treatment.

### 2.2. Statistical analysis

Statistical analyses were performed in Statistica v13 (TIBCO Software Inc.). For analyses of the psychological scales, the drop in the value of each subscale after 12 weeks of pharmacological treatment was investigated. The change of each of the subscales was obtained by subtracting the value of a given subscale after 12 weeks from the value of the same subscale at admission. The obtained values represent the improvement of the patient’s condition on each scale.

The normal distribution was verified using the Shapiro–Wilk test. The comparison of continuous variables between two groups was performed with the Student’s *t*-test in case of normal distribution or Mann–Whitney test in case of non-normal distribution of the parameter in each group. For correlation analysis of continuous variables, correlation and regression analyses were performed.

In order to address the question of whether changes in PANSS subscales after 12 weeks of pharmacotherapy were dependent on immunological parameters, stepwise linear regression analyses were performed, for each of the PANSS subscales independently. Other blood-derived parameters were also included in order to correct for clinically important information. As modeled (dependent) parameter, the value of the change in the PANSS subscale of interest was used. Independent parameters included the given PANSS subscale value at admission, medication dose, BMI, number of neutrophils (103/μl), number of lymphocytes (103/μl), CRP serum level (mg/L), complement C3 (g/L), complement C4 (g/L), fT3 (free triiodothyronine) (pmol/L), fT4 (free thyroxin) (pmol/L), natrium (mmol/L), creatinine (μmol/L), glucose (mmol/L), cortisol (μg/dl), HDL (mmol/L), LDL (mmol/L), triglycerides (mmol/L) (all values measured at admission).

The statistical significance cut-off value was α = 0.05. Where appropriate, Bonferroni corrections for multiple comparisons were applied.

## 3. Results

### 3.1. The inflammation indicator CRP itself does not correlate with PANSS subscale changes after treatment

C-reactive protein levels have repeatedly been shown to be altered in patients with schizophrenia as compared to healthy individuals (6, 17, 36–38). In our study group, none of the five subscales at the time of admission correlated significantly with CRP concentrations (Table 1a), nor did the change of the subscales after pharmacological treatment when no other parameters were taken into account (Table 1b). However, the STAI – state parameter was significantly higher in patients with CRP level 5 mg/L (which is the upper limit of the reference value for the healthy population) or above than in patients having this parameter in the reference range (*P* = 0.004).

**TABLE 1A:** Statistical association between elevated C-reactive protein levels at admission and the patients’ psychological state at admission.

CRP ≥5.0 mg/L	*P*-value
PANSS_pos	0.20
PANSS_neg	0.07
PANSS_dis	0.20
PANSS_exc	0.04
PANSS_emo	0.05
STAI_state	0.004*
BDI	0.31
Calgary	0.54

*Significant at α with Bonferroni correction 0.006. Statistical tests: Mann–Whitney for Calgary scale, Student’s *t*-test for remaining scales.

**TABLE 1B:** Statistical association between elevated C-reactive protein levels at admission and change in the patients’ psychological state after 12 weeks of unified pharmacological treatment.

CRP ≥5.0 mg/L	*P*-value
PANSS_pos change	0.66
PANSS_neg change	0.41
PANSS_dis change	0.68
PANSS_exc change	0.22
PANSS_emo change	0.01
STAI_state change	0.07
BDI change	0.49
Calgary change	0.56

α with Bonferroni correction: 0.006. Statistical tests: Mann–Whitney for PANSS_neg and PANSS_exc changes, Student’s *t*-test for remaining scales.

After 12 weeks of treatment, none of the changes in scales correlated with CRP, when no other factors were corrected for, which means that an elevated CRP level at admission alone is not indicative of the expected efficacy of treatment.

### 3.2. PANSS subscale changes correlate with their baseline values

**FIGURE 1:**
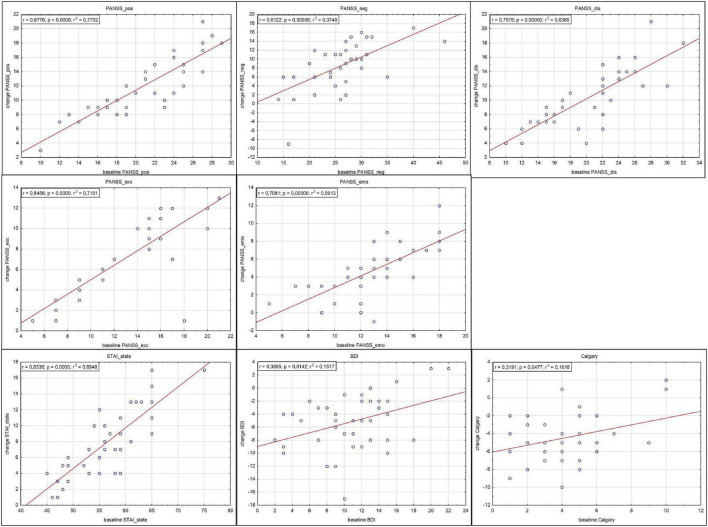
Correlations between subscale drops (indicating improvement) after 12 weeks of treatment and subscale values at admission.

Changes in the subscales noted after 12 weeks of treatment were positively dependent on the value of the given subscale at baseline, i.e., in general, the higher the baseline value, the bigger the observed drop of the scale after treatment, at week 12 (Figure 1).

### 3.3. Changes in PANSS subscales do not depend on medication dose

The change in none of the measures of mental status correlated with drug dose at admission. Corrections for baseline values were taken. *P*-values determined by regression analysis were 0.05, 0.75, 0.88, 0.36, and 0.87 for correlations of medication dose and changes in PANSS_pos, PANSS_neg, PANSS_dis, PANSS_exc, and PANSS_emo, respectively, corrected for the appropriate baseline value. This means that higher values at admission were characterized by a greater decrease in values after 12 weeks of pharmacological treatment, independently of the amount of the pharmacological substance the patients received.

### 3.4. Immunological parameters influence the changes in PANSS subscales

As seen in Table 2a, the change in the PANSS_pos subscale was impacted by the C4 complement level at admission and when this parameter was included in the analysis, also the dose of pharmacological treatment was significantly correlated with those values, with correction on the baseline PANSS_pos value. The baseline value of PANSS_pos had the largest impact on the drop of this value after 12 weeks, while C4 complement and medication dose had a similar impact strength (as indicated by β – see Table 2a). Both C4 complement level and medication dose were negatively correlated with the change in PANSS_pos, which means that simultaneously occurring lower values of those parameters corresponded to larger drops in the PANSS_pos scale after 12 weeks of pharmacological treatment. After removal of the medication dose from the model, also C4 complement becomes insignificant. The corrected *R*^2^ value indicates the superiority of this more complex model over the model with only the baseline PANSS_pos value. This means that for PANSS_pos, the simultaneous consideration of baseline PANSS_pos score, C4 level and the implemented pharmacological treatment dose are best predictive for the change in PANSS_pos after 12 weeks of treatment.

**TABLE 2A:** Results of stepwise linear regression analyses for subscales of the Positive and Negative Syndrome Scale.

Adj. *R*^2^ = 0.816; *P* (model) = 2⋅10^−12^									
	*b* (PANSS_pos change)	*b* SE	*P*-value	–95.00% CI	+95.00% CI	β	β SE	–95.00% CI	+95.00% CI
Intercept	-2.113	1.429	0.15	-5.024	0.797				
C4 complement	-10.914	4.179	0.01*	-19.427	-2.401	-0.199	0.076	-0.353	-0.044
Chlorpromazine	-0.012	0.004	0.009*	-0.021	-0.003	-0.231	0.084	-0.402	-0.062
PANSS_pos	0.847	0.070	2⋅10–13*	0.703	0.991	1.033	0.086	0.857	1.208

Modeling of PANSS_pos scale change after 12 weeks of pharmacological treatment.

*Statistically significant at α = 0.05.

All parameters present values at the time of admission.

When testing the influence of different parameters on PANSS_dis subscale change prediction, this scale depended not only on its value at admission but additionally on the inflammation-indicative parameter CRP, glucose, and creatinine levels at admission, with an adjusted *R*^2^ value indicating the superiority of this model over the model with baseline PANSS_dis scale values only (Table 2b). Also in this case, the greatest influence on the scale change value was provided by the baseline value of PANSS_dis and the correlation with the immunological factor was negative, which means that the lower the serum CRP level at admission, the bigger the drop of the PANSS_dis scale after 12 weeks of pharmacological treatment.

**TABLE 2B:** Results of stepwise linear regression analyses for subscales of the Positive and Negative Syndrome Scale.

Adj. *R*^2^ = 0.743; *P* (model) = 1⋅10^−9^									
	*b* (PANSS_dis change)	*b* SE	*P*-value	–95.00% CI	+95.00% CI	β	β SE	–95.00% CI	+95.00% CI
Intercept	-11.881	3.250	<0.001*	-18.509	-5.253				
CRP	-0.2313	0.079	0.006*	-0.392	-0.070	-0.263	0.090	-0.446	-0.080
Glucose	1.360	0.541	0.01*	0.257	2.463	0.220	0.087	0.042	0.399
Creatinine	0.062	0.023	0.01*	0.016	0.109	0.239	0.088	0.060	0.418
PANSS_dis	0.587	0.066	5⋅10–10*	0.452	0.721	0.793	0.089	0.610	0.975

Modeling of PANSS_dis scale change after 12 weeks of treatment.

*Statistically significant at α = 0.05.

All parameters present values at the time of admission.

Similarly to the above scale, PANSS_neg change was also positively dependent on its baseline value, glucose and creatinine levels but – differently than in the case of PANSS_dis, also negatively on the thyroid functioning indicator fT3 instead of inflammation parameters (Table 2c). In this case, all the parameters in the model had a similar influence strength.

**TABLE 2C:** Results of stepwise linear regression analyses for subscales of the Positive and Negative Syndrome Scale.

Adj. *R*^2^ = 0.639; *P* (model) = 2⋅10^−7^									
	*b* (PANSS_neg change)	*b* SE	*P*-value	–95.00% CI	+95.00% CI	β	β SE	–95.00% CI	+95.00% CI
Intercept	-16.871	6.176	0.01*	-29.468	-4.274				
fT3	-1.306	0.540	0.02*	-2.406	-0.205	-0.267	0.110	-0.492	-0.042
Glucose	2.898	0.849	0.002*	1.167	4.629	0.350	0.103	0.141	0.560
Creatinine	0.123	0.036	0.002*	0.049	0.196	0.353	0.104	0.141	0.565
PANSS_neg	0.320	0.091	0.001*	0.133	0.506	0.394	0.113	0.164	0.624

Modeling of PANSS_neg scale change after 12 weeks of treatment.

*Statistically significant at α = 0.05.

All parameters present values at the time of admission.

PANSS_exc and PANSS_emo were not dependent on immunological parameters and were most strongly influenced by their values at admission (Tables 2d, e).

**TABLE 2D:** Results of stepwise linear regression analyses for subscales of the Positive and Negative Syndrome Scale.

Adj. *R*^2^ = 0.765; *P* (model) = 2⋅10^−11^									
	*b* (PANSS_exc change)	*b* SE	*P*-value	–95.00% CI	+95.00% CI	β	β SE	–95.00% CI	+95.00% CI
Intercept	-6.129	1.590	<0.001*	-9.364	-2.894				
Creatinine	0.069	0.022	0.003*	0.025	0.114	0.276	0.087	0.098	0.453
PANSS_exc	0.620	0.073	7⋅10–10*	0.472	0.767	0.747	0.087	0.569	0.925

Modeling of PANSS_exc scale change after 12 weeks of treatment.

*Statistically significant at α = 0.05.

All parameters present values at the time of admission.

**TABLE 2E:** Results of stepwise linear regression analyses for subscales of the Positive and Negative Syndrome Scale.

Adj. *R*^2^ = 0.562; *P* (model) = 5⋅10^−7^									
	*b* (PANSS_emo change)	*b* SE	*P*-value	–95.00% CI	+95.00% CI	β	β SE	–95.00% CI	+95.00% CI
Intercept	-0.168	1.948	0.93	-4.132	3.795				
HDL	-1.954	0.812	0.02*	-3.606	-0.303	-0.278	0.115	-0.513	-0.043
PANSS_emo	0.578	0.103	3⋅10–6*	0.369	0.787	0.649	0.115	0.414	0.884

Modeling of PANSS_exc scale change after 12 weeks of treatment.

*Statistically significant at α = 0.05.

All parameters present values at the time of admission.

The remaining scales did not reveal significant correlations for immunological parameters (and are therefore not shown). The change in STAI_state and BDI were dependent only on their initial values (adj. *R*^2^ = 0.686 and adj. *R*^2^ = 0.126, respectively). The correlation of the STAI_state with CRP was not significant for the change of this value after treatment. The change after treatment of the Calgary scale, which is a measure of depression level in schizophrenia patients, was modeled to be dependent only on the number of neutrophils at admission and not on the value at admission (not shown). However, the low value of the coefficient of determination for this model (adj. *R*^2^ = 0.086) indicates a low predictive value of this parameter for the change in the Calgary scale after treatment.

## 4. Discussion

In this study we show that, for schizophrenia patients, inexpensive objective markers may be obtained by testing immune and metabolic parameters from the patients’ blood, and may be added at a low cost to the standard care of schizophrenia patients in order to predict the outcome of pharmacological treatment. To our knowledge, our study is the first that provides evidence for the reasonability of such an attitude.

In general, our investigation identifies that unified medical treatment for 12 weeks improved the patients’ mental health, as measured by psychiatric scales. The improvement was higher when higher values of the psychiatric scales were present at admission.

Only changes in the subscales PANSS_pos and PANSS_dis after 12 weeks of unified pharmacological treatment depended on inflammation parameters, in addition to the initial values of those scales. Those immunological parameters were C4 complement at admission (with correction for medication dose) for PANSS_pos, and CRP level at admission for PANSS_dis. Both subscales represent the significant disruption of thought processes and perception of reality. In contrast, the scales representing negative symptoms like emotional and social withdrawal, depressive mood, hostility etc., did not depend on immune parameters. For both PANSS_pos and PANSS_dis, lower values of immunological parameters corresponded to larger drops in the corresponding scale after 12 weeks of pharmacological treatment. This is in agreement with literature data on other immune parameters. For example, it has been shown that patients having low concentrations of serum IL-2 or IL-8 at baseline showed greater improvement than patients presenting with higher IL-2 or IL-8 concentrations in the serum at baseline (40). It has previously been shown that neutrophils, monocytes, and CRP are in general increased in schizophrenia patients vs. controls at baseline. CRP correlated with the PANSS-P score at baseline in first-episode psychosis patients and the amelioration of positive symptoms as a result of treatment correlated with lower neutrophil levels or CRP values (36). Also in another study, in the first episode of schizophrenia, the levels of non-specific inflammation markers in the blood (WBS, CRP, erythrocytes sedimentation rate, and granulocytes from the leukocyte formula) were high in the subpopulation of patients, with the tendency toward normalization of inflammation parameters after a 4-week antipsychotic treatment (41). CRP has also previously been associated with cognitive impairment severity in schizophrenia patients but not with the severity of psychiatric symptoms (37). However, in the cited work, psychiatric symptoms were analyzed using the basic positive, negative, or general PANSS symptoms. On the contrary, in our study, we used subscales, as indicated by Marder et al. (27). The advantage of those subscales over the traditionally determined P (positive), N (negative), G (general), and T (total) is that those subscales are obtained based on principal components and are therefore linearly uncorrelated with each other, which allows for their independent interpretation. Each of the subscales represents a different area of symptoms. In our study group, none of the five subscales at the time of admission correlated significantly with CRP concentrations, nor did the change of the subscales after pharmacological treatment, when analyzed independently. However, the STAI – state parameter was significantly higher in patients with CRP level 5 mg/L or above than in patients having this parameter in the reference range. We additionally show that when the baseline value of the PANSS_dis subscale is included as a confounding factor, higher CRP levels at baseline may be related to a worse prognosis in the improvement after treatment of the subscale of PANSS related to disorganized thoughts. At the same time, the CRP value at admission has no significant impact on the change of other subscales, neither when analyzed alone nor together with confounding factors.

In the case of the subscale representing negative symptoms, the free thyroid hormone T3 was significant, together with levels of glucose and creatinine, which are markers of the patients’ metabolism. A correlation of negative symptoms with thyroid status can be found in previous literature. For example, in the study of Barbero et al. anti-thyroid antibodies correlated with a more severe phenotype, which was characterized by increased negative symptoms and poorer functioning in early psychotic patients (39). In the case of scales representing excitation and negative emotions, in our study, their changes after treatment were predominantly dependent on the initial value of the given scale but also on metabolic parameters.

The correlation of inflammation parameters with positive PANSS scales at admission, as well as of fT3 with PANSS_neg at admission has been shown by our team in a previous work (42). Here, we show that those parameters are significant also for the decline in those values after pharmacological treatment. In summary, we found that for the PANSS subscales PANSS_pos and PANSS_dis, representing excitation and disorganization of thought processes, the immunological parameters C4 and CRP, respectively, are modifying parameters for the outcome of pharmacological treatment. For negative symptoms, fT3, glucose and creatinine levels were significant modifiers, while scales representing excitation and negative emotions, were influenced by the parameters creatinine and HDL, respectively.

Measurement of mental parameters on subjective scales based on interviews with the patients remains the standard for clinical assessment of patients with mental illness. The clinical phenotype is particularly variable for patients with schizophrenia, who are therefore vulnerable to polypragmasia. The introduction of the suggested changes into the standard clinical care would result in the addition, at a low financial cost, of objective markers of a patient’s chance of improving specific areas of their functioning following pharmacological treatment. The addition of laboratory markers, i.e., CRP, C4, and metabolic parameters, to the standard of psychiatric care during a patient’s admission to a psychiatric ward is a cost of only around €100–160 per patient (Supplementary Table 1). This cost assessment is based on commercially available diagnostic tests. Performing these assays in a hospital laboratory for internal use will incur an even lower cost. Considering the cost of chronic pharmacotherapy with neuroleptics, the complications generated by this treatment and their management (including cardiometabolic disfunctions, i.e., metabolic syndrome, obesity, tachycardia, dyslipidemia, myocardial infarction, stroke; and hematological disorders, e.g., agranulocytosis) and the cost of monitoring the patient’s clinical status, the addition of inexpensive and objective markers obtained by diagnostic laboratory tests to the primary care of psychiatric patients with a heterogeneous spectrum of symptoms seems reasonable.

## 5. Conclusion

Concluding, in this study, we provide practical evidence that basic immunological and metabolic markers may be of value in the therapeutics course of patients with schizophrenia. Our results show how easily accessible diagnostic values obtained at patient admission may be used in predicting the outcome of given aspects of the clinical schizophrenia picture. Moreover, we provide a cost-effective research framework and laboratory work for frontline clinicians interested in the relationship of the immune system and metabolism with schizophrenia. Validation of those results on additional patient cohorts may lead to the implementation of such predictors in everyday practice.

## Data availability statement

The original contributions presented in this study are included in the article/Supplementary material, further inquiries can be directed to the corresponding author.

## Ethics statement

The studies involving human participants were reviewed and approved by the Bioethics Committee of the Jagiellonian University, consent number: 122.6120.23.2016 from the 23rd June 2016. Written informed consent to participate in this study was provided by the participants’ legal guardian/next of kin.

## Author contributions

AS wrote the first draft of the manuscript and counted the results and interpreted them to the constructed statistical model. NŚ, MS, MP, and PD recruited patients and conducted the clinical and biochemical evaluation. WK, TK, and MP provided critical revision of the manuscript and important intellectual contributions. All authors contributed to the article and approved the submitted version.

## References

[1] Müller N Riedel M Ackenheil M Schwarz M. The role of immune function in schizophrenia: an overview. *Eur Arch Psychiatry Clin Neurosci.* (1999) 249(Suppl):62–8. 10.1007/pl00014187 10654111

[2] Na K Jung H Kim Y. The role of pro-inflammatory cytokines in the neuroinflammation and neurogenesis of schizophrenia. *Prog Neuropsychopharmacol Biol Psychiatry.* (2014) 48:277–86. 10.1016/j.pnpbp.2012.10.022 23123365

[3] Brown A Derkits E. Prenatal infection and schizophrenia: a review of epidemiologic and translational studies. *Am J Psychiatry.* (2010) 167:261–80. 10.1176/appi.ajp.2009.09030361 20123911 PMC3652286

[4] Khandaker G Zimbron J Lewis G Jones P. Prenatal maternal infection, neurodevelopment and adult schizophrenia: a systematic review of population-based studies. *Psychol Med.* (2013) 43:239–57. 10.1017/S0033291712000736 22717193 PMC3479084

[5] Khandaker G Zimbron J Dalman C Lewis G Jones P. Childhood infection and adult schizophrenia: a meta-analysis of population-based studies. *Schizophr Res.* (2012) 139:161–8. 10.1016/j.schres.2012.05.023 22704639 PMC3485564

[6] de Picker L Fransen E Coppens V Timmers M de Boer P Oberacher H Immune and neuroendocrine trait and state markers in psychotic illness: decreased kynurenines marking psychotic exacerbations. *Front Immunol.* (2019) 10:2971. 10.3389/fimmu.2019.02971 32010121 PMC6978914

[7] Müller N Ulmschneider M Scheppach C Schwarz M Ackenheil M Möller H COX-2 inhibition as a treatment approach in schizophrenia: immunological considerations and clinical effects of celecoxib add-on therapy. *Eur Arch Psychiatry Clin Neurosci.* (2004) 254:14–22. 10.1007/s00406-004-0478-1 14991374

[8] Jeppesen R Christensen R Pedersen E Nordentoft M Hjorthøj C Köhler-Forsberg O Efficacy and safety of anti-inflammatory agents in treatment of psychotic disorders – a comprehensive systematic review and meta-analysis. *Brain Behav Immun.* (2020) 90:364–80. 10.1016/j.bbi.2020.08.028 32890697

[9] Kubistova A Horacek J Novak T. Increased interleukin-6 and tumor necrosis factor alpha in first episode schizophrenia patients versus healthy controls. *Psychiatr Danub.* (2012) 24(Suppl. 1):S153–6. 22945211

[10] Dunjic-Kostic B Jasovic-Gasic M Ivkovic M Radonjic NV Pantovic M Damjanovic A Serum levels of interleukin-6 and tumor necrosis factor-alpha in exacerbation and remission phase of schizophrenia. *Psychiatr Danub.* (2013) 25:55–61. 23470607

[11] He S Zhang L Yu S Yu W Yu Y Huang J Association between tumor necrosis factor-alpha (TNF-a) polymorphisms and schizophrenia: an updated meta-analysis. *Int J Psychiatry Clin Pract.* (2022) 26:294–302. 10.1080/13651501.2021.2009879 35188044

[12] Qing Y Xu L Cui G Sun L Hu X Yang X Salivary microbiome profiling reveals a dysbiotic schizophrenia-associated microbiota. *NPJ Schizophr.* (2021) 7:51. 10.1038/s41537-021-00180-1 34711862 PMC8553823

[13] Charles S Mogle J Piazza J Karlamangla A Almeida D. Going the distance: the diurnal range of cortisol and its association with cognitive and physiological functioning. *Psychoneuroendocrinology.* (2020) 112:104516. 10.1016/j.psyneuen.2019.104516 31805455 PMC6948931

[14] Mikulska J Juszczyk G Gawrońska-Grzywacz M Herbet M. HPA Axis in the pathomechanism of depression and schizophrenia: new therapeutic strategies based on its participation. *Brain Sci.* (2021) 11:1298. 10.3390/brainsci11101298 PMC8533829 34679364

[15] Yuan X Wang S Shi Y Yang Y Zhang Y Xia L Pro-inflammatory cytokine levels are elevated in female patients with schizophrenia treated with clozapine. *Psychopharmacology (Berl).* (2022) 239:765–71. 10.1007/s00213-022-06067-y 35080634

[16] Leboyer M Godin O Terro E Boukouaci W Lu C Andre M Immune signatures of treatment-resistant schizophrenia: a fondamental academic centers of expertise for schizophrenia (FACE-SZ) study. *Schizophr Bull Open.* (2021) 2:sgab012. 10.1093/schizbullopen/sgab012 34901861 PMC8650073

[17] Ma J Zhang Y Huang Z Liu X Lv L Li Y. Relationship between curative effect and serum inflammatory factors level in male patients with first-episode schizophrenia treated with olanzapine. *Front Psychiatry.* (2021) 12:782289. 10.3389/fpsyt.2021.782289 34955927 PMC8695839

[18] Zhang Q He H Cao B Gao R Jiang L Zhang X Analysis of cognitive impairment in schizophrenia based on machine learning: interaction between psychological stress and immune system. *Neurosci Lett.* (2021) 760:136084. 10.1016/j.neulet.2021.136084 34174347

[19] Bessler H Cohen-Terica D Djaldetti M Sirota P. The effect of ibuprofen on cytokine production by mononuclear cells from schizophrenic patients. *Folia Biol (Praha).* (2017) 63:13–9. 28374670 10.14712/fb2017063010013

[20] Serafini G Parisi V Aguglia A Amerio A Sampogna G Fiorillo A Specific inflammatory profile underlying suicide risk? systematic review of the main literature findings. *Int J Environ Res Public Health.* (2020) 17:2393. 10.3390/ijerph17072393 32244611 PMC7177217

[21] Serafini G Adavastro G Canepa G de Berardis D Valchera A Pompili M The efficacy of buprenorphine in major depression, treatment-resistant depression and suicidal behavior: a systematic review. *Int J Mol Sci.* (2018) 19:2410. 10.3390/ijms19082410 30111745 PMC6121503

[22] Hadjulis M Margariti M Lazaridou M Aggelidis G Fotopoulos V Markaki L Clinical guidelines for the management of schizophrenia: pharmacological and psychological interventions (III). *Psychiatriki.* (2019) 29:303–15. 10.22365/jpsych.2018.294.303 30814040

[23] Taylor D Paton C Paton C. *The Maudsley Prescribing Guidelines.* Boca Raton, FL: CRC Press (2009). 10.3109/9780203092835

[24] Danivas V Venkatasubramanian G. Current perspectives on chlorpromazine equivalents: comparing apples and oranges! *Indian J Psychiatry.* (2013) 55:207. 10.4103/0019-5545.111475 23825865 PMC3696254

[25] Kay S Fiszbein A Opler L. The positive and negative syndrome scale (PANSS) for schizophrenia. *Schizophr Bull.* (1987) 13:261–76. 10.1093/schbul/13.2.261 3616518

[26] Suzuki T. Which rating scales are regarded as “the standard” in clinical trials for schizophrenia? A critical review. *Psychopharmacol Bull.* (2011) 44:18–31. 22506437 10.64719/pb.4069 PMC5044554

[27] Marder S Davis J Chouinard G. The effects of risperidone on the five dimensions of schizophrenia derived by factor analysis. *J Clin Psychiatry.* (1997) 58:538–46. 10.4088/JCP.v58n1205 9448657

[28] Spielberger C Gorsuch R Lushene R. *Manual for the State-Trait Anxiety Inventory.* Palo Alto, CA: Consulting Psychologists Press (1970).

[29] Wrześniewski M Sosnowski A Jaworowska T Fecenec D. *Inwentarz Stanu i Cechy Lêku (Polska Adaptacja STAI).* Warszawa: Pracownia Testów Psychologicznych Polskiego Towarzystwa Psychologicznego (2011). p. 1–115.

[30] Beck A Steer R Brown G. *BDI-II, Beck Depression Inventory?: Manual.* San Antonio, TX: Psychological Corp (1996).

[31] Łojek E Stańczak J. *BDI-II Inwentarz Depresji Becka – Wydanie Drugie.* Warszawa: Pracownia Testów Psychologicznych Polskiego Towarzystwa Psychologicznego (2019).

[32] Addington D Addington J Schissel B. A depression rating scale for schizophrenics. *Schizophr Res.* (1990) 3:247–51. 10.1016/0920-9964(90)90005-R 2278986

[33] Szafrański T Jarema M Ruzikowska A Chóma M Kukulska D. Reliability and validity of the polish version of the calgary depression rating scale. *Schizophr Res.* (2000) 41:47–8. 10.1016/S0920-9964(00)90405-5

[34] Addington D Addington J Matickatyndale E. Specificity of the calgary depression scale for schizophrenics. *Schizophr Res.* (1994) 11:239–44. 10.1016/0920-9964(94)90017-5 8193062

[35] Müller M Kienzle B Dahmen N. Depression, emotional blunting, and akinesia in schizophrenia. *Eur J Health Econ.* (2002) 3:s99–103. 10.1007/s10198-002-0114-9 15609162

[36] Steiner J Frodl T Schiltz K Dobrowolny H Jacobs R Fernandes B Innate immune cells and c-reactive protein in acute first-episode psychosis and schizophrenia: relationship to psychopathology and treatment. *Schizophr Bull.* (2020) 46:363–73. 10.1093/schbul/sbz068 31504969 PMC7442383

[37] Dickerson F Stallings C Origoni A Boronow J Yolken R. C-reactive protein is associated with the severity of cognitive impairment but not of psychiatric symptoms in individuals with schizophrenia. *Schizophr Res.* (2007) 93:261–5. 10.1016/j.schres.2007.03.022 17490859

[38] Yan J Chen Y Ju P Gao J Zhang L Li J Network association of biochemical and inflammatory abnormalities with psychiatric symptoms in first-episode schizophrenia patients. *Front Psychiatry.* (2022) 13:834539. 10.3389/fpsyt.2022.834539 35273531 PMC8901486

[39] Barbero J Palacín A Serra P Solé M Ortega L Cabezas Á Association between anti-thyroid antibodies and negative symptoms in early psychosis. *Early Interv Psychiatry.* (2020) 14:470–5. 10.1111/eip.12873 31529601

[40] Zhang X Zhou D Cao L Zhang P Wu G Shen Y. Changes in serum interleukin-2, -6, and -8 levels before and during treatment with risperidone and haloperidol: relationship to outcome in schizophrenia. *J Clin Psychiatry.* (2004) 65:940–7. 10.4088/jcp.v65n0710 15291683

[41] Stefanović V Mihajlović G Nenadović M Dejanović S Borovcanin M Trajković G. The effect of antipsychotic drugs on nonspecific inflammation markers in the first episode of schizophrenia. *Vojnosanit Pregl.* (2015) 72:1085–92. 10.2298/vsp140526016s 26898032

[42] Śmierciak N Szwajca M Popiela T Bryll A Karcz P Donicz P Redefining the cut-off ranges for tsh based on the clinical picture, results of neuroimaging and laboratory tests in unsupervised cluster analysis as individualized diagnosis of early schizophrenia. *J Pers Med.* (2022) 12:247. 10.3390/jpm12020247 35207735 PMC8874519

