# Supplementary material for: Immunological routine laboratory parameters at admission influence the improvement of positive symptoms in schizophrenia patients after pharmacological treatment
Source: Front Psychiatry. 2023 Mar 22;14:1082135. doi: 10.3389/fpsyt.2023.1082135 (PMC10073498; doi:10.3389/fpsyt.2023.1082135)
Supplement: Supplementary file 1 [file Table_1.DOCX]

Supplementary Table 1. Commercial costs of diagnostic laboratory tests used in the study, based on online-available offers of commercial laboratories located in different EU countries.

| **Laboratory test** | **cost in Euro** |
| --- | --- |
| Full blood count | 5-15 |
| CRP, high sensitive | 10-30 |
| Complement C3+C4 | 10-20 |
| fT3 | 16-20 |
| fT4 | 16-20 |
| Natrium | 2-5 |
| Creatinine | 2-5 |
| Glucose (fasting blood level) | 2-5 |
| Cortisol | 15-20 |
| Lipid profile | 15-20 |
| **Total cost (Euro) per patient** | **93-160** |
